# Echocardiography: a help in the weaning process

**DOI:** 10.1186/cc9076

**Published:** 2010-06-22

**Authors:** Vincent Caille, Jean-Bernard Amiel, Cyril Charron, Guillaume Belliard, Antoine Vieillard-Baron, Philippe Vignon

**Affiliations:** 1 Réanimation médicale, CHU Ambroise Paré, 9 avenue Charles-de-Gaulle, 92104 Boulogne, France; 2 Faculté de Paris Ile-de-France Ouest, Université de Versailles Saint Quentin en Yvelines, 78000 Versailles, France; 3 Réanimation Polyvalente, CHU de Limoges, 2 avenue Martin Luther King, 87042 Limoges, France; 4 Centre d'investigation clinique CIC-P 0801, 2 avenue Martin Luther King, 87042 Limoges, France; 5 Université de Limoges, 87000 Limoges, France

## Abstract

**Introduction:**

To evaluate the ability of transthoracic echocardiography (TTE) to detect the effects of spontaneous breathing trial (SBT) on central hemodynamics and to identify indices predictive of cardiac-related weaning failure.

**Methods:**

TTE was performed just before and at the end of a 30-min SBT in 117 patients fulfilling weaning criteria. Maximal velocities of mitral E and A waves, deceleration time of E wave (DTE), maximal velocity of E' wave (tissue Doppler at the lateral mitral annulus), and left ventricular (LV) stroke volume were measured. Values of TTE parameters were compared between baseline (pressure support ventilation) and SBT in all patients and according to LV ejection fraction (EF): >50% (n = 58), 35% to 50% (n = 30), and <35% (n = 29). Baseline TTE indices were also compared between patients who were weaned (n = 94) and those who failed (n = 23).

**Results:**

Weaning failure was of cardiac origin in 20/23 patients (87%). SBT resulted in a significant increase in cardiac output and E/A, and a shortened DTE. At baseline, DTE was significantly shorter in patients with LVEF <35% when compared to other subgroups (median [25^th^-75^th ^percentiles]: 119 ms [90-153]; vs. 187 ms [144-224] vs. 174 ms [152-193]; *P *< 0.01) and E/E' was greater (7.9 [5.4-9.1] vs. 6.0 [5.3-9.0] vs. 5.2 [4.7-6.0]; *P *< 0.01). When compared to patients who were successfully weaned, those patients who failed exhibited at baseline a significantly lower LVEF (36% [27-55] vs. 51% [43-55]: *P *= 0.04) and higher E/E' (7.0 [5.0-9.2] vs. 5.6 [5.2-6.3]: *P *= 0.04).

**Conclusions:**

TTE detects SBT-induced changes in central hemodynamics. When performed by an experienced operator prior to SBT, TTE helps in identifying patients at high risk of cardiac-related weaning failure when documenting a depressed LVEF, shortened DTE and increased E/E'. Further studies are needed to evaluate the impact of this screening strategy on the weaning process and patient outcome.

## Introduction

Weaning patients from the ventilator remains a crucial issue. In 2,500 patients included in 6 large randomized trials, the incidence of weaning failure, which is defined as a failed spontaneous breathing trial (SBT) or the need for a re-intubation in the 48 hours following extubation, reached 31% [1]. Weaning failure remains a clinically relevant challenge because it may result in significant morbidity (prolonged duration of mechanical ventilation, re-intubation) and may influence mortality [2,3].

Even if its actual incidence is unknown, cardiac dysfunction is a leading cause of weaning failure [1]. Breathing in the context of weaning was described as a physical exercise [4]. The abrupt cessation of positive pressure ventilation increases venous return and left ventricular (LV) afterload [5], decreases LV compliance [6], and may even induce cardiac ischemia [7]. All these factors tend to increase LV filling pressure [6,8], and may subsequently result in cardiogenic pulmonary edema. Right heart catheterization has long been used in this clinical setting to detect an increase of the pulmonary artery occlusion pressure (PAOP) [6,9]. Nevertheless, PAOP may be difficult to precisely measure in the presence of large swings in intra-thoracic pressure, as observed in spontaneously breathing patients with increased inspiratory efforts [10].

Although echocardiography allows the noninvasive assessment of cardiac function and LV filling pressures, its clinical value in the setting of ventilator weaning has yet to be determined. In this prospective, descriptive, bicentric study, we sought to evaluate the ability of transthoracic echocardiography (TTE) to detect the effects of SBT on central hemodynamics and to potentially identify indices that could help predicting weaning failure from cardiac origin.

## Materials and methods

This prospective study was conducted from January 2006 to August 2007 in the ICU of two University hospitals. No change of standard care was introduced for the need of this study, which was therefore accepted as a descriptive study by the Clinical Research Ethics Committee of the Société de Réanimation de Langue Française. Written informed consent was waived but all patients or their next of kin were informed.

### Study population

All patients mechanically ventilated for more than 48 hours were eligible if they fulfilled the following weaning criteria [1]: resolution of the acute episode for which the patient was placed on ventilator, pressure support ventilation (PS/positive end-expiratory pressure (PEEP)), adequate cough, absence of excessive tracheo-bronchial secretion, stable cardiovascular status (heart rate ≤120/min, systolic blood pressure higher that 90 mmHg and lower than 160 mmHg), adequate oxygenation (partial pressure of artierial oxygen (PaO_2_)/fraction of inspired oxygen (FiO_2_) ≥150, PEEP ≤8 cmH_2_O), adequate pulmonary function (respiratory rate ≤35 breathes/min, tidal volume > 5 mL/kg, no significant respiratory acidosis), no sedation or stable neurological status. Patients were ineligible if they were not in sinus rhythm or had atrioventricular conduction abnormalities, if they had a pace-maker, if an apical four-chamber view was not possible to obtain, or if the intensivist experienced in echocardiography was unavailable. In all patients, the simplified acute physiology score (SAPS) II [11] was calculated. As a standard of care in participating centers, dobutamine was initiated in the presence of hypotension (systolic blood pressure < 90 mmHg) associated with decreased LV ejection fraction (EF) at a starting dose of 5 μg/kg/min and without exceeding 10 μg/kg/min. Norepinephrine was indicated in the presence of hypotension with preserved LVEF and no sign of preload-dependence, and the dose was tailored to reach a mean blood pressure of more than 65 mmHg. During the study period, vasoactive drugs were not initiated and the dose of ongoing infusion remained constant.

### Study protocol

The SBT was performed over a 30-minute period using a T-piece while the patient was in a semi-recumbent position (45°), as recommended [1]. SBT failure was defined as the need to connect the patient back to the ventilator prior to its completion due to at least one of the following reasons: agitation and anxiety or depressed mental status, cyanosis, percutaneous oxygen saturation (SpO_2_) above 90%, respiratory rate of more than 35 breathes/min, heart rate above 150 beats/min or cardiac arrhythmia, systolic blood pressure above 180 mmHg or below 90 mmHg. When the SBT was successful, the planned extubation was performed. The attending physician in charge of the patient did not have access to TTE results.

All patients were included at the time of their first SBT. Failure to wean a patient from the ventilator was defined as a failed SBT or the need for a re-intubation within 48 hours following extubation [1]. In the latter case, medical records were reviewed by the medical staff to identify the cause of weaning failure and to specifically confirm or confidently exclude an underlying cardiogenic pulmonary edema based on clinical and radiological criteria. No access to the TTE report was allowed.

Heart rate, systolic and diastolic blood pressure, respiratory rate, pulse oxymetry, five-lead electrocardiographic tracing and level of consciousness were closely monitored during the SBT, as widely advocated [1].

### Doppler echocardiography

TTE was performed by experienced operators with a level 3 competence in echocardiography [12]. The respiratory cycle was displayed on the screen of the ultrasound machine (airway pressure or plethysmography) to precisely identify end-expiration for standardized measurements. TTE was initially performed in a patient under pressure support prior to the deconnection from the ventilator on a T-piece, and subsequently at the end of the SBT (i.e., before the planned extubation or before the reconnection to the ventilator required by the deterioration of respiratory status). In each participating center, the same experienced operator performed the two TTE studies. Imaging sequences were digitally stored for off-line measurements.

In the apical four-chamber view, LVEF was measured using the modified Simpson's rule and both the right ventricular (RV) and LV end-diastolic areas (EDA) were measured to calculate the RVEDA/LVEDA ratio. A dilated right ventricle was defined by a ratio of more than 0.6 [13]. Color Doppler mapping was used to detect the presence of a relevant mitral regurgitation and to assess its severity semi-quantitatively (minor versus moderate-to-severe). Positioning pulse-wave Doppler at the tip of the mitral valve leaflets, we also recorded LV inflow velocities. Maximal flow velocity during early diastole (E wave) and during atrial systole (A wave) was measured, and the E/A ratio was computed. The deceleration time of the E wave (DTE) was measured in extending the deceleration slope from the peak wave velocity to the zero-velocity baseline [14]. By using pulse-wave tissue Doppler at the lateral portion of the mitral annulus, we measured the maximal velocity of its displacement during early diastole (E' wave), and the E/E' ratio was computed [15]. Finally, LV stroke volume was measured using the Doppler method applied at the level of LV outflow tract and cardiac output was calculated [16]. All measurements were performed in triplicate at end-expiration and averaged.

### Statistical analysis

Statistical calculations were performed using the StatView 5 (SAS Institute Inc., Cary, NC, USA). Continuous echocardiographic variables were expressed as medians with 25^th ^to 75^th ^percentiles. Values of TTE parameters were compared between baseline (pressure support ventilation) and the SBT using the Wilcoxon paired test. This comparison was performed in the overall study population and in three subsets of patients according to the value of LVEF at baseline (under pressure support ventilation): LVEF below 30%, LVEF between 30% and 50%, and LVEF above 50%. Comparison of echocardiographic variables in PS/PEEP according to the value of LVEF was performed using a one-way analysis of variance. Statistical significance was considered for a two-tailed *P *value less than 0.05.

In one of the participating centers, inter-and intra-observer variability for the measurement of E and A maximal velocity, DTE, and maximal E' velocity are 1% and 2%, 3% and 2%, 13% and 7%, and 5% and 2%, respectively [17]. In the other participating center, the inter-and intra-observer variability for the measurement of E/A and DTE was 4% and 3%, and 6% and 6%, respectively (VC, AVB).

## Results

Of 142 eligible patients, 25 were excluded because of the absence of adequate apical four-chamber view (n = 7), atrial fibrillation (n = 13), paced cardiac rhythm (n = 4), and agitation precluding image acquisition (n = 1). Accordingly, 117 patients were studied (71 men, 46 women, age: 63 (58 to 67) years [median (25^th ^to 75^th ^percentile)]; SAPS II: 53 (47 to 58)). Sixteen patients (14%) had pre-existing chronic obstructive pulmonary disease (COPD). Reasons for intubation were acute respiratory failure (n = 20), severe sepsis or septic shock (n = 42), cardiogenic shock (n = 40), and neurological disorders and stroke (n = 15). Duration of mechanical ventilation was 5 (5 to 6.5) days and overall mortality in the ICU reached 10% (Table 1). Before the SBT, levels of pressure support and PEEP were 12 (10 to 12) cmH_2_O and 3 (3 to 4) cmH_2_O, respectively. Twelve percent of patients had already received diuretics in the 48 hours preceding the SBT.

**Table 1:** Characteristics of the study population

	All patients (n = 117)
Age (years)	63 (58-67)
Gender (men/women)	71/46
Height (cm)	168 (166-170)
Weight (kg)	75 (71-79)
SAPS II	53 (47-58)
Pre-existing cardiac disease, n (%)	35 (30)
Hypertension, n (%)	26 (33)
Pre-existing COPD, n (%)	16 (14)
Dobutamine infusion, n (%)	10 (9)
Dose μg/kg/min	4.8 (3.5-5.0)
Epinephrine-norepinephrine, n (%)	15 (15)
Dose μg/kg/min	0.11 (0.08-0.14)
Diuretic therapy before SBT, n (%)	14 (12)
Duration of mechanical ventilation (days)	5.0 (5.0-6.5)
Mortality, n (%)	11 (10)

COPD, chronic obstructive pulmonary disease; SBT, spontaneous breathing trial; SAPS II, simplified acute physiology score II [11].

SBT was unsuccessful in 11 patients (9%) for weaning-induced pulmonary edema. Among the 106 extubated patients, 12 (11%) were re-intubated for post-extubation cardiogenic pulmonary edema (n = 9), weakness and increased airway secretions (n = 2), and for stridor in the remaining patient. Overall, the incidence of weaning failure was 20% (23/117 patients), and related to a cardiac origin in 20 of 23 patients (87%). No episode of cardiac ischemia was documented, both during SBT and respiratory distress requiring reintubation. Three patients exhibited atrial fibrillation and two patients had marked sinus tachycardia (> 140 bpm) during the SBT, three of whom failing the weaning process.

During the SBT, cardiac output and systolic arterial pressure significantly increased (Table 2). The increase in cardiac output was related to SBT-induced tachycardia because LV stroke volume remained unchanged. Mitral E/A significantly increased and E/E' tended to increase without reaching statistical significance, while DTE significantly decreased (Table 2). No change in RV size was observed, as reflected by the same median RVEDA/LVEDA ratio measured during PS/PEEP and SBT (Table 2). No moderate-to-severe mitral regurgitation was observed.

**Table 2:** Echocardiographic findings in the 117 patients during pressure support ventilation (PS/PEEP) and the spontaneous breathing trial (SBT)

	PS/PEEP	SBT	*P *value
**HR (/min)**	95 (90-99)	98 (91-103)	0.00001
**SAP (mmHg)**	138 (132-143)	145 (136-152)	0.019
**CO (L/min)**	5.8 (5.2-6.2)	6.0 (5.4-6.7)	0.004
**SV (mL)**	62 (57-67)	64 (58-70)	0.5
**E/A**	0.94 (0.82-1.05)	1.00 (0.88-1.15)	0.003
**DTE (ms)**	168 (150-187)	147 (132-160)	0.00001
**E/E'**	5.9 (5.4-6.4)	6.5 (5.7-7.2)	0.16
**RVEDA/LVEDA**	0.47 (0.44-0.50)	0.47 (0.44-0.50)	0.79

CO, cardiac output; SV, stroke volume; E/A, ratio of maximal mitral E wave and A wave velocities; DTE, deceleration time of mitral E wave; HR, heart rate; LVEDA, left ventricular end-diastolic area; RVEDA, right ventricular end-diastolic area; SAP, systolic arterial pressure.

Weaning failure was observed in 10 of 58 patients (17%) with an LVEF above 50%, in 4 of 30 patients (13%) with an LVEF between 35 to 50%, and in 9 of 29 patients (31%) with an LVEF below 35% (*P *< 0.05). At baseline (PS/PEEP), E/A was similar between groups, whereas DTE was significantly lower and E/E' significantly higher in patients with a LVEF below 35% (Table 3). During SBT, E/A significantly increased and DTE significantly decreased solely in patients with an LVEF of 50% or less. E/E' tended to increase during SBT in patients with LVEF of 35% or more without reaching statistical significance, whereas it remained elevated with no further increase in patients with a LVEF below 35% (Table 3). In patients who failed to be weaned from the ventilator due to a cardiogenic pulmonary edema (n = 20), both the median E/A and E/E' ratios increased significantly from PS/PEEP to SBT (1.05 (0.64 to 1.81) vs. 1.19 (0.71 to 3.18): *P *< 0.01 and 8.0 (4.8 to 9.3) vs. 8.7 (5.3 to 10.8): *P *< 0.05, respectively), whereas median DTE decreased from 105 ms (87 to 185) to 90 ms (75 to 133; *P *< 0.05).

**Table 3:** Doppler echocardiographic findings according to baseline left ventricular ejection fraction (recorded under pressure support ventilation)

		LVEF > 50% (n = 58)	LVEF 50-35% (n = 30)	LVEF < 35% (n = 29)
**E/A**				
	PS/PEEP	0.97 (0.81-1.09)	0.82 (0.74-1.0)	1.0 (0.78-1.47)
	SBT	0.99 (0.85-1.20)	0.89* (0.76-1.32)	1.0* (0.86-1.89)
**DTE** (ms)				
	PS/PEEP	174 (152-193)	187 (144-224)	119**^¶^** (90-153)
	SBT	163 (155-181)	140* (112-177)	96* (80-137)
**E/E'**				
	PS/PEEP	5.2 (4.7-6.0)	6.0 (5.3-9.0)	7.9**^¶^** (5.4-9.1)
	SBT	5.8 (4.9-6.6)	6.9 (4.9-9.3)	7.8 (6.7-9.7)

DTE, deceleration time of mitral E wave; LVEF, left ventricular ejection fraction; PS/PEEP, pressure support ventilation; SBT, spontaneous breathing trial.

* *P *< 0.05 versus PS/PEEP.

**^¶ ^***P *< 0.01 in PS/PEEP versus other groups.

In patients who could not be weaned from the ventilator, LVEF was significantly lower and E/E' was significantly higher than in those who underwent weaning success (Table 4). Despite a significantly higher heart rate, patients who failed to be weaned had a lower cardiac output due to a substantial reduction of LV stroke volume. E/A was similar between the two subsets of patients whereas DTE was shortened in the case of weaning failure, although not reaching statistical significance (Table 4).

**Table 4:** Patients' characteristics prior to SBT, according weaning success or failure

	Weaning success (n = 94)	Weaning failure (n = 23)	*P *value
**SAP (mmHg)**	139 (133-147)	132 (115-149)	0.28
**HR (bpm)**	92 (86-97)	110 (95-120)	**0.007**
**SV (mL)**	63 (57-70)	60 (39-66)	0.06
**CO (L/min/m^2^)**	5.8 (5.2-6.3)	5.4 (3.3-6.5)	0.19
**LVEF (%)**	51 (43-55)	36 (27-55)	**0.04**
**E/A**	0.94 (0.82-1.03)	0.88 (0.68-1.65)	0.7
**DTE (ms)**	170 (150-189)	138 (98-195)	0.07
**E/E'**	5.6 (5.2-6.3)	7.0 (5.0-9.2)	**0.038**
**RVEDA/LVEDA**	0.47 (0.44-0.51)	0.48 (0.43-0.52)	0.99

CO, cardiac output; DTE, deceleration time of mitral E wave; HR, heart rate; LVEDA, left ventricular end-diastolic area; LVEF, LV ejection fraction; RVEDA, right ventricular end-diastolic area; SAP, systolic arterial pressure; SV, left ventricular stroke volume.

## Discussion

In the present study, we showed that TTE can accurately depict changes in central hemodynamics induced by SBT and potentially select patients at high risk of cardiac-related weaning failure. In our patients, SBT resulted in a significant increase in heart rate and in cardiac output well-reflecting the greater work of breathing which has been compared with a true exercise [4]. We also observed a rise in systolic arterial pressure, which was consistent with greater LV afterloading. Although not statistically significant, the increase in E/E' during the SBT is in keeping with an increase in LV filling pressure induced by the shift from positive pressure ventilation to spontaneous breathing. More significant, both the increase of E/A and shortened DTE during the SBT suggest that altered LV diastolic properties may also potentially contribute to the rise in LV filling pressure [14]. Similar results were found by Ait-Oufella and colleagues [18] in 31 patients who were successfully weaned from the ventilator. In the present study, median RV/LV end-diastolic area was similar prior to and at the end of the SBT. This presumably reflects the absence of SBT-induced pulmonary hypertension in our study population, which comprised a low proportion of COPD patients.

Under PS/PEEP, E/A was not a discriminating Doppler parameter between subgroups of patients based on baseline LVEF. In contrast, DTE was significantly reduced in patients with severely depressed LV systolic function when compared with other subsets of patients, and E/E' ratio significantly increased with the deterioration of LV systolic function. ICU patients with LV systolic dysfunction who enter the process of weaning from the ventilator usually have underlying heart disease with associated LV diastolic dysfunction, reduced compliance and increased filling pressure [19]. In our patients with LVEF below 35%, shortened DTE presumably reflected underlying LV diastolic dysfunction, while the gradual increase in E/E' ratio across the three subsets of patients was consistent with the progressive elevation of cardiac filling pressure which was associated with the deterioration of LV compliance [20]. Interestingly, a statistically significant increase of E/A ratio and shortening of DTE during SBT was only observed in the subsets of patients with LV dysfunction. This suggests further deterioration in LV diastolic properties induced by SBT, which may be attributed to decreased LV compliance [6] or even silent cardiac ischemia [7], as previously suggested in ICU patients with associated LV systolic dysfunction [18]. In our patients with LVEF below 35%, median E/E' at baseline (under PS/PEEP) was as high as 7.9 and failed to further increase during the SBT. We previously showed in ventilated ICU patients that a lateral E/E' ratio above 8.0 predicted a PAOP of more than 18 mmHg with a 83% sensitivity and a 88% specificity [21]. Similarly, Lamia and colleagues recently reported that the conjunction of an E/A ratio above 0.95 and E/E' ratio above 8.5 at the end of the SBT in a selected population of patients difficult to wean allowed predicting a PAOP of 18 mmHg or more with a 82% sensitivity and a 91% specificity [22]. Interestingly, no significant change in E/E' was observed during SBT in our patients, regardless of LVEF. In 102 ICU patients, Mekontso-Dessap and colleagues reported that circulating brain natriuretic peptide (BNP), a biomarker correlated with LV filling pressure, failed to increase at the end of a 60-minute SBT [23], whereas Grasso and colleagues showed that NT-proBNP increased only in those patients who developed acute cardiac dysfunction during the SBT [24].

When compared with patients who successfully completed the weaning process, patients under PS/PEEP who failed had significantly lower LVEF and higher E/E', and tended to have shorter DTE. In addition, the subset of patients who failed to be weaned from the ventilator exhibited a significant increase of both E/A and E/E' and a shortened DTE at the time of the SBT, when compared with PS/PEEP. In keeping with our results, Mekontso-Dessap and colleagues found that circulating BNP was significantly increased in patients under PS/PEEP who finally failed the weaning process, as a result of an overloaded LV [23]. TTE appears ideally suited to routinely screen patients at risk of weaning-related pulmonary edema (e.g., chronic obstructive pulmonary disease, heart failure) prior to the SBT. Patients with a severely decreased LVEF (< 35%) should be considered at high risk of cardiac-related weaning failure, particularly when exhibiting shortened DTE and elevated E/E'. These TTE indices are simple yet robust and fairly reproducible [17]. This screening strategy could potentially help the intensivist to better select patients for tailored therapy as an attempt to facilitate the weaning process (e.g., diuretics, control of systolic blood pressure, non invasive pressure support ventilation after extubation). In the present study, a large proportion of patients with predisposing heart or lung disease would have been eligible for such a clinical approach which still remains to be validated.

In this clinical setting, TTE must be performed by an experienced intensivist with an advanced level in critical care echocardiography [25], because image acquisition and interpretation is frequently challenging. Intensivists with competence for basic level critical care echocardiography have the ability of qualitatively assessing LVEF, but are not adequately trained to precisely evaluate LV diastolic properties and filling pressures [25]. Such training requirement is undoubtedly a substantial limitation of this TTE-based approach. Only four of our patients (5%) were excluded because of poor echogenicity because all examinations have been performed by the same experienced operators. In contrast, Grasso and colleagues reported in COPD patients a 26% rate of inadequate four-chamber view [24]. This apparent discrepancy is presumably explained by the markedly lower proportion of COPD patients in our study and by the measurement of LVEF which requires an optimal visualization of the endocardial border in the study by Grasso and colleagues. We only used pulse-wave Doppler during the SBT which remains possible to acquire and accurate even in the presence of suboptimal two-dimensional image quality.

Our study has several limitations. First, the physiologically interesting changes in E/A and DTE during SBT cannot be used in clinical practice to select patients at high risk of cardiac-related weaning failure. Indeed, the fairly large inter-patient variability observed in absolute variations of Doppler parameters induced by the SBT precluded the determination of a clinically useful threshold value. However, we found that the echocardiographic profile during PS/PEEP was more relevant to select those patients at high risk of cardiac-related weaning failure. Second, we could not clearly identify using TTE whether LV diastolic dysfunction or increased LV filling pressure was the leading cause of weaning failure in our patients. As these two entities are closely linked [19,20], a combined effect is presumably operant in the setting of weaning process from the ventilator. Third, up to 15% of our patients had received diuretics during the 48 hours preceding the SBT. This may have introduced a relevant bias of selection in our study and led to soften the effects of SBT on central hemodynamics, especially in patients with severely reduced LV function. This may also have reduced the proportion of weaning pulmonary edema and might explain the lower incidence of weaning failure in our series (20%) when compared with previous larger cohorts of patients with a mean 31% rate of weaning failure [1]. Fourth, silent myocardial ischemia cannot be confidently excluded in our patients because a conventional 12-lead ECG was not recorded prior to and at the end of SBT. Fifth, we purposely excluded patients with non sinus rhythm because Doppler indices are more challenging to precisely measure in this setting. Nevertheless, DTE and E/E' have also been validated to evaluate LV filling pressure in patients with atrial fibrillation [26,27]. Finally, we did not investigate the additional value of combining a biological marker to echocardiography to better evaluate the cardiovascular system during weaning.

## Conclusions

TTE appears as a sensitive noninvasive method which accurately detects changes in central hemodynamics induced by the SBT. When performed by an experienced operator prior to SBT (during pressure support ventilation), TTE helps the attending physician to identify patients at high risk of weaning failure, when documenting a depressed LVEF, shortened DTE and increased E/E'. Further studies are needed to evaluate the impact of this screening strategy on the weaning process and patient outcome.

## Key messages

• TTE accurately reflects changes in central hemodynamics induced by SBT.

• Those changes include an increase in mitral Doppler E/A ratio and shortening in E wave deceleration time, as a reflection of increased LV filling pressure and potential diastolic dysfunction, and are more pronounced in patients with decreased LVEF.

• In patients examined prior to SBT, TTE findings predictive of weaning failure were: a decreased LVEF, a shortened mitral E wave deceleration time and an elevated E/E' ratio.

• In patients who failed to be weaned from the ventilator due to a cardiogenic pulmonary edema (n = 20), median E/A and E/E' ratios increased significantly from pressure support ventilation to SBT, whereas median DTE significantly decreased.

• No significant SBT-induced alteration in RV size has been observed.

## Abbreviations

BNP: brain natriuretic peptide; COPD: chronic obstructive pulmonary disease; DTE: deceleration time of mitral Doppler E wave; EDA: end-diastolic area; EF: ejection fraction; FiO2: fraction of inspired oxygen; LV: left ventricle; PaO2: partial pressure of arterial oxygen; PAOP: pulmonary artery occlusion pressure; PEEP: positive end-expiratory pressure; PS/PEEP: pressure support ventilation; RV: right ventricle; SAPS: simplified acute physiology score; SBT: spontaneous breathing trial; TTE: transthoracic echocardiography.

## Competing interests

The authors declare that they have no competing interests.

## Authors' contributions

VC, JBA, CC, GB, AVB, PV participated to the elaboration of the study project, the enrollment of patients and performance of echocardiographic examinations, and they participated to data analysis. VC, AVB and PV contributed to the preparation of the manuscript.
